# Supplementary material for: Srebp-1c/Fgf21/Pgc-1α Axis Regulated by Leptin Signaling in Adipocytes—Possible Mechanism of Caloric Restriction-Associated Metabolic Remodeling of White Adipose Tissue
Source: Nutrients. 2020 Jul 10;12(7):2054. doi: 10.3390/nu12072054 (PMC7400870; doi:10.3390/nu12072054)
Supplement: Supplementary file 1 [file nutrients-12-02054-s001.pdf]

Figure S1

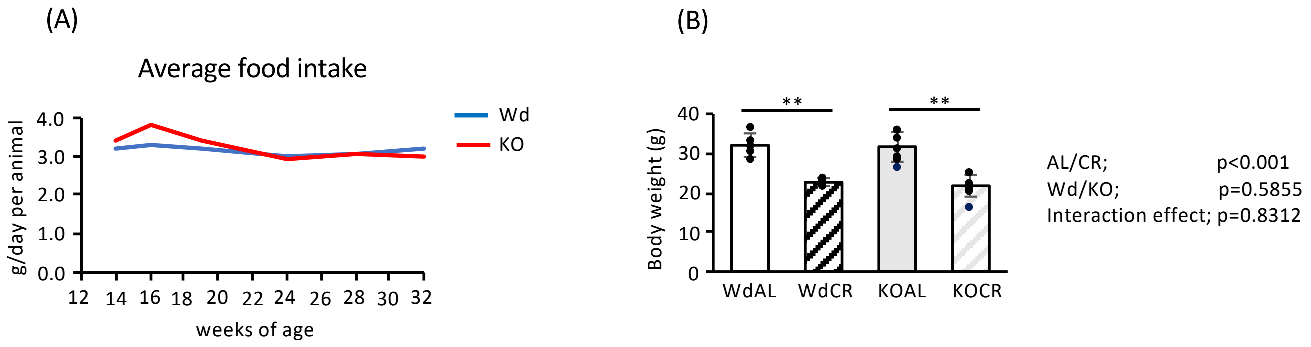

Figure S1. Time course measurement of food intake of Wd and KO mice (A), and body weight of four groups at 10 months of age (n=5-8) (B). Values are means ± SDs, **P < 0.01 vs. AL, according to Tukey's test after two-way ANOVA.

Figure S2

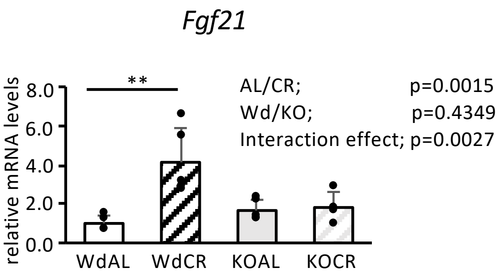

Figure S2. The effects of Srebp-1c KO on the expression of *Fgf21* in the WAT of mice on a C57Bl/6-129S6 background. The mRNA expression levels of *Fgf21* in WAT were measured using real-time RT-PCR and were normalized to *Tbp* expression (n=4-5). Values are means ± SDs, **P < 0.01 vs. AL, according to Tukey's test after two-way ANOVA.

Figure S3

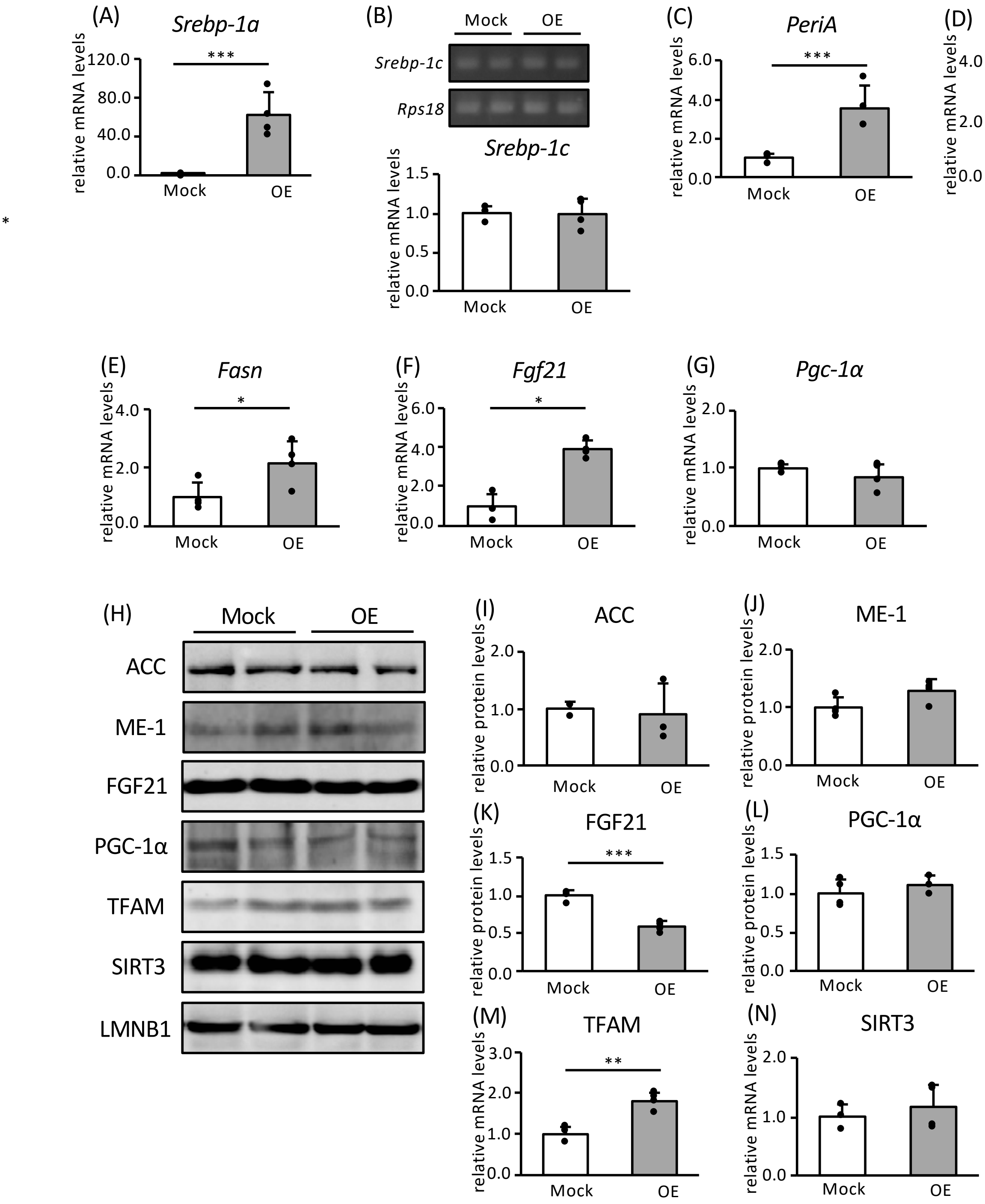

Figure S3. The effects of SREBP-1a overexpression on the expression of genes and proteins involved in FA biosynthesis and mitochondrial biogenesis in mature 3T3-L1 adipocytes. Control and SREBP-1a OE preadipocytes were differentiated into mature adipocytes in four separate dishes for each phenotype. RNA was extracted and lysates were prepared from each dish. The mRNA expression levels of Srebp-1a (A), PeriA (C), Adipoq (D), Fasn (E), Fgf21 (F), and Pgc-1a (G) were determined using real-time RT-PCR and normalized to Rps18 expression (n=4). (B) Representative image of ethidium bromide-stained gels showing the fluorescence of RT-PCR products corresponding to Srebp-1c. Semiquantitative analysis was performed and the data were normalized to Rps18 expression (n=4). (H) Representative immunoblot images showing the expression levels of proteins involved in FA biosynthesis and mitochondrial biogenesis. Quantitative analysis was performed using a chemiluminescence method. The protein expression levels of ACC (I), ME-1 (J), FGF21 (K), PGC-1α (L), TFAM (M), and SIRT3 (N) are shown as the relative intensity of the indicated protein divided by that of LMNB1 as an internal control (n=4). Values are means ± SDs, $^*P < 0.05$, $^{**}P < 0.01$, $^{***}P < 0.001$ vs. control, according to Student's t-test.
